# Improving accuracy for cancer classification with a new algorithm for genes selection

**DOI:** 10.1186/1471-2105-13-298

**Published:** 2012-11-13

**Authors:** Hongyan Zhang, Haiyan Wang, Zhijun Dai, Ming-shun Chen, Zheming Yuan

**Affiliations:** 1 Hunan Provincial Key Laboratory of Crop Germplasm Innovation and Utilization, Changsha, 410128, China; 2 College of Bio-safety Science and Technology, Hunan Agricultural University, Changsha, 410128, China; 3 Department of Statistics, Kansas State University, Manhattan, KS, 66506, USA; 4 USDA-ARS and Department of Entomology, Kansas State University, Manhattan, KS, 66506, USA; 5 College of Information Science and Technology, Hunan Agricultural University, Changsha, 410128, China

## Abstract

**Background:**

Even though the classification of cancer tissue samples based on gene expression data has advanced considerably in recent years, it faces great challenges to improve accuracy. One of the challenges is to establish an effective method that can select a parsimonious set of relevant genes. So far, most methods for gene selection in literature focus on screening individual or pairs of genes without considering the possible interactions among genes. Here we introduce a new computational method named the Binary Matrix Shuffling Filter (BMSF). It not only overcomes the difficulty associated with the search schemes of traditional wrapper methods and overfitting problem in large dimensional search space but also takes potential gene interactions into account during gene selection. This method, coupled with Support Vector Machine (SVM) for implementation, often selects very small number of genes for easy model interpretability.

**Results:**

We applied our method to 9 two-class gene expression datasets involving human cancers. During the gene selection process, the set of genes to be kept in the model was recursively refined and repeatedly updated according to the effect of a given gene on the contributions of other genes in reference to their usefulness in cancer classification. The small number of informative genes selected from each dataset leads to significantly improved leave-one-out (LOOCV) classification accuracy across all 9 datasets for multiple classifiers. Our method also exhibits broad generalization in the genes selected since multiple commonly used classifiers achieved either equivalent or much higher LOOCV accuracy than those reported in literature.

**Conclusions:**

Evaluation of a gene’s contribution to binary cancer classification is better to be considered after adjusting for the joint effect of a large number of other genes. A computationally efficient search scheme was provided to perform effective search in the extensive feature space that includes possible interactions of many genes. Performance of the algorithm applied to 9 datasets suggests that it is possible to improve the accuracy of cancer classification by a big margin when joint effects of many genes are considered.

## Background

Classification of cancer tissue samples based on microarray expression data is of great interest in recent years. This was driven by biomedical applications to differentiate cancerous tissue samples from normal samples as well as different tumor subtypes. Though many methods have been recently developed, further improvement in classification accuracy is needed before molecular methods can be used to replace laborious histological approaches. An obvious challenge for effective classification is that the number of samples is much smaller than the number of available features. There are two directions to tackle the challenge. Some studies focus on how to design or create better classifiers with a given sets of features. Examples are SVM [1], Top Scoring Pair (TSP) [2], k-Top Scoring Pair (k-TSP) [3], and Hyper-box Enclosure (HBE) [4]. A common feature of these methods is that they depend critically on prescreening of genes such as through comparing the absolute value of a T-statistic. The other direction is to seek ways to reduce the dimensionality of the feature space and select the informative genes for effective classification with new or existing classifiers. Efforts in this direction include, for example, Prediction Analysis of Microarrays (PAM, [5]), individual-gene-ranking methods by evaluating the discriminating power of classes (see [6,7], and the references therein), redundant gene filtering through correlation analyses [8,9] or Based Bayes error Filter (BBF) [10].

Individual gene-ranking methods perform gene preselection through a univariate criterion function to provide a list of top ranked genes. However, the combination of top ranked genes through individual gene-ranking may not produce a top ranked combination of genes because individual ranking tends to ignore the redundancy and interaction among genes. As a result, such methods tend to have low power when the co-regulation of multiple genes or pathways during tumor progression is not fully utilized. An example is shown in Table 1 of this paper, in which the combination of six genes selected yields high accuracy for classification of the colon cancer data [11]. However, two out of the six genes have p-values greater than 0.9. These two genes have extremely low chance to be selected by individual gene-ranking methods. Recently, Chopra et al. [12] proposed to use doublets made from gene pair combinations as inputs to cancer classification algorithms. The rationale behind the doublets is that biomolecular pathways may be stronger biomarkers for cancer as compared to individual genes [13]. It is shown by Chopra et al. [12] that upon using doublets, classification accuracy of several classification algorithms were consistently improved across different datasets compared to the same algorithms with the same number of single genes.

**Table 1:** Selected genes from original colon dataset after screening by t-test

**Accession Number**	**p-value of t-test**	**p-value of paired t-test**
T48041	0.90237	0.01289
M19311	0.95889	1.2E-08
T51023	0.00121	3.0E-09
D63874	0.00798	0.00006
X57206	0.11898	5.4E-07
T57882	0.18106	0.00006

The paired t-test was conducted according to the method described in section "Importance ordering and significance of the selected genes".

It is more realistic and promising to extend the doublets method to include multiple genes instead of only gene pairs since pathways often involve several crucial genes. For example, it has been reported that colorectal carcinoma is developed from the accumulation of genetic alterations, including chromosomal instability, gene mutations, and epigenetic abnormality after initiated by inactivation of the adenomatous polyposis coli tumour-suppressor pathway in a cell within the colon [14,15]. The doublet method can describe the co-regulation patterns of two genes (either up-up, up-down, or down-down) with simple operations such as summation, difference, and multiplication. However, for a pathway involving three or more genes, such simple operations are not sufficient to describe the exponentially increasing number of patterns. In such cases, unknown and possibly complex interactions among genes will add additional features to the already N-P hard problem due to the small sample size and large number of features. If all genes and their interactions at all levels (two-way and higher-way interactions) were to be considered in the search space, the dimension of the search space is ultra-high. Consider the colon cancer microarray data [11] for an example, the feature subset search space contains 2^2000^ features when all interactions are included while the sample size is only 62. The colon cancer data contains the least number of genes among those we considered. The other cancer microarray datasets contain at least 7000 genes and the sample size is mostly much less than 100. Including interactions among genes essentially makes the feature subset search space have infinite dimension.

Available variable selection methods often fall into one of the three categories: filtering approaches, wrapper approaches, and embedded methods, where the latter class combines advantages of filters and wrappers. A filtering approach assesses the relevance of a feature subset without consulting with a classification algorithm, meanwhile a wrapper approach searches the optimal feature set that maximized the classification performance defined in terms of an evaluation function (such as cross-validation accuracy). It has been well accepted that wrapper approaches tend to provide better classification accuracy than the same algorithms with variable selection through filtering approaches (see [16] and the references therein). One limitation with the wrapper methods is that the search of optimal feature subsets for different classification algorithms needs to be conducted separately. Consequently the feature subset selected by one algorithm does not generalize well to other algorithms [16,17]. Another limitation of the traditional wrapper methods is that the estimation of the evaluation function in feature subset selection may cause an overfitting problem when the sample size is small. Note that a sample size of 100 with less than 50 features is deemed small in [16]. The sample sizes in the data setting of this article are extremely small in presence of the near infinite dimensional feature search space. Therefore, a simple application of traditional wrapper methods will incur a serious overfitting problem in current settings that leads to overly optimistic evaluation of the function and poor classification accuracy of the test data. A third limitation with the wrapper methods is that they need almost prohibitive computation by exhaustively searching through all possible combinations of gene sets. Hill-climbing (greedy search, or steepest ascent) and best-first search are such examples. They are only applicable when the number of features is small. Partial search schemes, such as sequential forward selection, backward elimination, sequential floating backward elimination and random search, were often employed. A drawback with the partial search is that the algorithm may converge to a locally optimal solution instead of the global optimum. How to perform the search in the space of feature subsets has been studied for many years. Seeking effective searching schemes in wrapper methods for data with a large number of features remains to be an active topic. Consistent with our goal of considering gene interactions in the search space is a claim by Cover and Campenhout [18] that even for multivariate normally distributed features, no greedy search procedure that selects one feature at a time can find the best feature subset of a desired size; even an algorithm that adds the best pair and removes the worst single feature can fail.

Embedded methods use internal information of the classification model to perform feature selection. Support Vector Machine Recursive Feature Elimination (SVM-RFE), known as an excellent feature ranking algorithm, is one of the embedded methods. In SVM-RFE algorithm, the objective function J is half the L^2^ norm of the weight vector. In linear kernel SVM, the weight vector can be calculated explicitly. In each iteration, the elimination of the feature with the least squared weight will cause the least effect on J [19-21]. Therefore, the weight vector is adopted as ranking criterion. To improve the efficiency of the algorithm, more features can be eliminated at each step [22]. Recently, Liu et al. [23] extended SVM-RFE to RBF kernel based on Recursive Feature Elimination (SVM-RBF-RFE) by expanding nonlinear RBF kernel into its Maclaurin series to calculate the weight vector.

Ensemble methods are a class of popular methods that combine the effort of both classifier building and feature selection. An ensemble method uses multiple classifiers to produce a learner system. Many mechanisms have been proposed for the creation of ensemble of classifiers in the literature. These include using different subsets of training data with a single learning technique, using different learning methods, or using different training parameters with a single learning method. One popular ensemble algorithm for datasets with a large number of features is the random subspace method [24], which represents a class of learning ensembles of weak classifiers to achieve good prediction accuracy. A random subspace method generates a large number of weak classifiers each trained on randomly chosen low dimensional subspace of the original input space. The ensemble output is obtained through majority voting or aggregation techniques (cf. [25]). Random space search methods effectively reduce the curse of dimensionality problem and are known to improve weak classifiers. Random forest with the tree classifier, one of the random subspace search methods, has been demonstrated to have comparable performance to other classification methods including Diagonal Linear Discriminant Analysis (DLDA), K nearest neighbor (KNN), and SVM. Open issues related to random subspace search methods include the conditions under which ensemble outperforms an individual classifier and how to determine a suitable ensemble size for a task with given computational requirements in terms of memory size and CPU time [26]. Other ensemble methods including bagging [27] which employs majority voting over results from a large number of bootstrap samples, and boosting [28-30] which construct classifiers on weighted versions of the training set which depends on previous classification results. Bagging changes the distribution of the data stochastically and boosting changes the distribution of the data set adaptively based on the performance of previously created learners. Skurichina and Duin [31] demonstrated usefulness of bagging, boosting and the random subspace method in linear discriminant analysis. See [26] for a more detailed review of different ensemble methods. An ensemble combing bagging, boosting, rotation forest and random subspace version of the same learning algorithm using a voting methodology was also proposed in [26].

**Figure 1:**
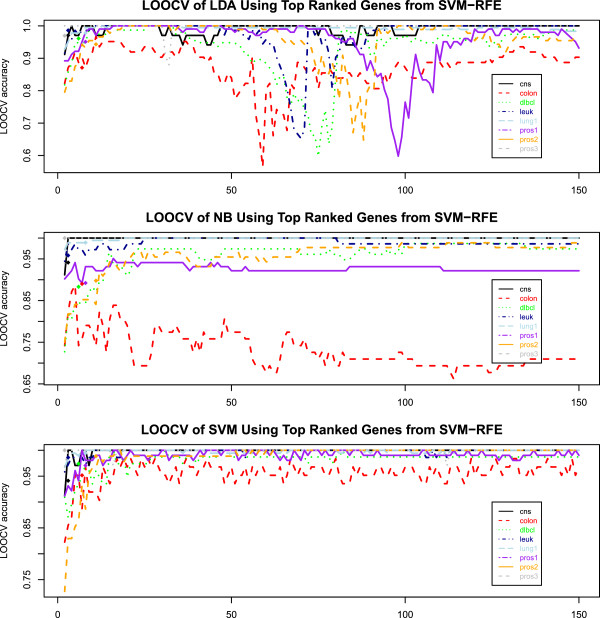
**Plot of LOOCV accuracy of LDA, NB, and SVM using *k *top ranked genes from SVM-RFE for *k *= 2, …, 150.** The accuracy of SVM in general increases as more genes are included in the model. The accuracies of LDA and NB do not show an increasing pattern suggesting that the gene ranking by SVM-RFE is SVM specific and may not generalize well to NB or LDA. The plotted curves assume the number of genes is known (oracle situation). Without knowing the number of genes to be used, additional variability will add to the LOOCV accuracy. The diamond-shaped points show the LOOCV accuracy of the LDA, NB, and SVM classifiers using the genes selected by BMSF.

There are some unsettled challenges associated with SVM-RFE and the random space search methods: (1) In SVM-RFE and some of the random space search methods, the number of variables to be selected or the subspace dimension size is often set to be a fixed known parameter that requires the user to supply a value. The SVM-RFE methods only provide a ranking of the features and rely on the user to specify the number of features to be selected. The weighted random subspace method by Li and Zhao [25] only considered the number of variables to be 10 in all their experiments. In practice, the number of informative features is unknown and need to be well estimated in order for the ensemble classifiers to have good performance. When the number of features used in the subspace ensemble is less than the true number that generated the response variable, the resulting classifiers may not capture the pattern. When the number of features used is more than the true one, the noisy features included would degrade the performance of the classifiers. How to reliably estimate the number of informative features remain to be a challenging problem. (2) The SVM-RFE top ranked genes do not generalize well to other algorithms. The top ranked genes from SVM-RFE may have poor classification performance when they are used with other classifiers. For instance, we obtained the list of ranked genes using the SVM-RFE algorithm (removing one feature at a time) on several cancer datasets. Then we obtained the LOOCV accuracy with the top k genes in each data set using the linear discriminant classifier (LDA) and the Naïve Bayes classifier (NB) for, k=2, …, 150 (see Figure 1). The LOOCV accuracy of the LDA classifier using the top ranked genes provided by SVM-RFE accuracy increases initially as the number of genes increases but then decreases and fluctuates a lot when the number of genes is between 50 and 100. In many cases, the accuracy falls below 70% for different cancer microarray data sets. The NB classifier applied to the colon cancer data even has an overall decreasing pattern for LOOCV accuracy as the number of top ranked genes from SVM-RFE increases.

In this article, we present a hybrid method to overcome some of the challenges mentioned above for ensemble and wrapper methods in the high dimensional feature selection with small sample sizes. That is, we consider how to avoid overfitting and provide effective search of informative genes in the infinite dimensional search space with limited resource (storage and speed of model training and optimization), how to incorporate gene interactions in the search scheme to provide high classification accuracy for multiple algorithms, and how to determine the number of informative genes. We named our method Binary Matrix Shuffling Filter (BMSF). BMSF is a data-driven guided random search algorithm implemented through binary matrix shuffling coupled with SVM to perform filtering. The method introduces intermediate data matrices with binary values to convert the high-dimensional feature space search into the optimization problem of support vector machine regression (SVR) with the response being the Matthew’s correlation coefficient and predictors being multiple factors each with two levels (see Section 3.1). Extensive search of the large dimensional feature space including gene interactions is made feasible by reducing the number of models trained and exploiting predications more often to take full advantage of SVR’s property of being time-consuming in training yet fast in prediction. As a result of less model training, the method smartly avoids the overfitting problem in small sample size and poor generalization drawback for the feature subset selection to other algorithms. The significance of our method includes: (1) The gene selection process considers the differentiating power of a gene conditional on the possibly nonlinear effects of other gene combinations. This allows complicated interactions among genes to be fully incorporated to reflect pathway changes during tumor progression. (2) BMSF often selects a relatively small number of genes that can accurately classify samples. This helps to achieve the goal of finding a minimal gene list to facilitate the search for new diagnostic tools and follow up study. (3) BMSF not only leads to improved LOOCV classification accuracy of SVM that was wrapped with the variable selection process, but also results in better performance for multiple classifiers including Naive Bayes (NB), linear discriminant analysis (LDA), quadratic discriminant analysis (QDA). (4) Using the genes selected by the BMSF, SVM, LDA, and QDA reached or exceeded the highest LOOCV accuracy reported in literature. This confirms that allowing interactions among features in the search space coupled with a manageable search scheme as in BMSF not only respect natural underlying bio-molecular reaction mechanisms but also provide better accuracy for biomarker selection.

## Results

Here we present the performance of the proposed algorithm on 9 benchmark binary class expression datasets all related to human cancers, including central nervous system, colorectal, diffuse large B-cell lymphoma, leukemia, lung, and prostate tumors. The sample size and number of genes in each dataset are summarized in Table 2.

**Table 2:** Summary of nine datasets used in our experiments

**Dataset**	**No. of Genes**	**No. of samples in class I**	**No. of samples in class II**	**Source**
CNS	7129	25(C)	9(D)	[32]
Colon	2000	40(T)	22(N)	[11]
DLBCL	7129	58(D)	19(F)	[33]
GCM	16063	190(C)	90(N)	[34]
Leukemia	7129	25(AML)	47(ALL)	[35]
Lung	12533	150(A)	31(M)	[36]
Prostate1	12600	52(T)	50(N)	[37]
Prostate2	12625	38(T)	50(N)	[38]
Prostate3	12626	24(T)	9(N)	[39]

### Final list of Informative genes

As mentioned in the introduction, many classifiers and feature selection methods preprocess the data by applying a univariate test (such as a t-test) to reduce the number of genes before conducting further refined procedures. The preprocessing briefly assesses the main effect of one gene without considering the effects of other genes. To be consistent with the literature and make it easy for comparison, we only report the selected genes after a preprocessing with the t-test and then applying our algorithm on the genes with *p*-value less than α=0.05. Table 3 summarizes the selected genes, some of which are new while others can also be found in the literature. For example, consider the three informative genes found for the leukemia data. X95735 (Zyxin) was found to play an important role in differentiating acute myelogenous leukemia (AML) and acute lymphoblastic leukemia (ALL) by multiple authors [35,40-43]. Zyxin is a zinc-binding phosphoprotein that concentrates along the actin cytoskeleton and at focal adhesions that enable cells to adhere to the extracellular matrix and at which protein complexes involved in signal transduction assemble [44]. Y07604 was reported as one of the top five genes with the highest selection frequency for classification of the leukemia data in Yang et al. [43]. High expression of the protein encoded by Y07604 is associated with poor prognosis and advanced stages in myelodysplastic syndrome which frequently transforms into AML [45]. D26156 was reported to be a discriminatory gene between AML and ALL in Golub et al. [35] and was also among the top ranking genes reported in Broberg [40] based on multiple gene ranking methods. Real time PCR measurement of D26156 was highly expressed from patients with AML [46]. The protein encoded by D26156 is a member of the large ATP-dependent chromatin remodeling complex SWI/SNF family of proteins, which have helicase and ATPase activities and are thought to regulate transcription of certain genes by altering the chromatin structure around those genes [47]. In addition, this protein can bind BRCA1, as well as regulate the expression of the tumorigenic protein CD44. It is reported in Medina et al. [48] that the protein encoded by D26156 is a bona fide tumor suppressor and a major factor in lung tumorigenesis. The accession number of the genes for other datasets and their putative functions are listed in the Additional file 1: Table S1.

**Table 3:** Summary of selected genes

**Dataset**	**Number of genes**	**Selected genes**
CNS	3	J03507, U00968, Y00757
Colon	7	Z50753, H67764, H17434, R88740, R36977, R81170, U14631
DLBCL	6	K03430_at, M37815_cds1_at, X51688_at, X76534_at, Z70723_at, M16652_at
GCM	32	S82075_at, U35048_at, U61374_at, U87964_at, U95090_at, U97188_at, X14445_at, X92715_at, M29610_at, M21642_at, M19267_s_at, M19878_at, Z50115_s_at, X93511_s_at, X56687_s_at, AA256220_at, AA334630_at, AA362708_at, D30921_at, M54994_f_at, R10529_at, R29657_at, W27827_at, W39573_at, Z49995_at-2, RC_AA100437_at, RC_AA210695_at, RC_AA252372_at, RC_AA278134_at, RC_AA281769_s_at, RC_AA405698_at, RC_AA489009_at
Leukemia	3	X95735_at, Y07604_at, D26156_s_at
Lung	8	1779_s_at, 33246_at, 36354_at, 38221_at, 40363_r_at, 540_at, 631_g_at, 885_g_at
Prostate1	8	33614_at, 38322_at, 36627_at, 38041_at, 41303_r_at, 1846_at,930_at, 829_s_at
Prostate2	11	31673_s_at, 35099_at, 37821_at, 40038_at, 41077_at, 36030_at, 37209_g_at, 38983_at, 33928_r_at, 38832_r_at, 33188_at
Prostate3	2	39364_s_at, 40546_s_at

### Leave-one-out cross-validation accuracy

**Figure 2:**
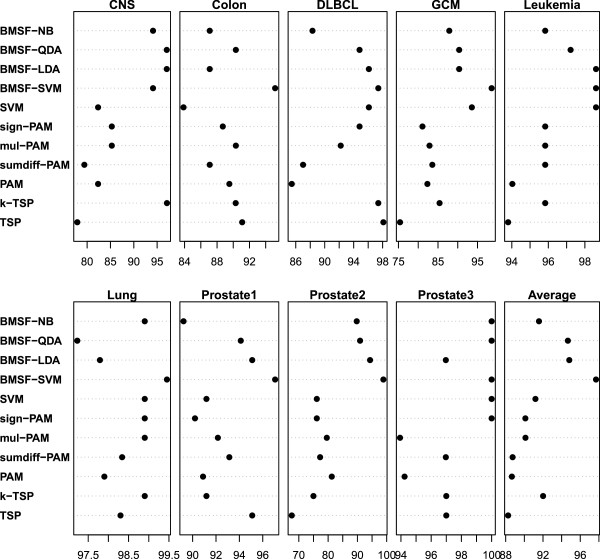
Comparison with top performance results reported in literature for nine cancer datasets.

The Leave-One-Out Cross-Validation (LOOCV) method was used to estimate the accuracy of classifiers. For each sample in the dataset, we used the rest of the samples in the dataset to serve as training data for model building and prediction for the class of this sample. For classifiers that have tuning parameters (such as SVM), the optimal parameters were first estimated with 5-fold CV using the training data and then used in the modeling. The classification accuracy of each dataset is the ratio of the number of the correctly classified samples to the total number of samples in that dataset, i.e., (True Positives+True Negatives)/total number of samples. To assess the generality of the selected informative genes, we also evaluate the performance of LDA, QDA, and NB using the selected genes in addition to SVM in LOOCV accuracy. The results along with some methods published in the recent literature are summarized in Figure 2 and the numerical values (denoted as BMSF-SVM/LDA/QDA/NB) are given in Additional file 1: Table S2.

Note that all methods in Additional file 1: Table S2 use all the samples in each data set to perform feature selection. As pointed out by a reviewer, the feature selection needs to use the training data if the main purpose is to estimate the generalization error for future samples. In such case, which genes are selected and the number of genes selected are not the focus of the study since each training data in the LOOCV procedure leads to a separate list of genes. In the end, there are as many lists of genes as the sample size. These different lists of genes are not helpful when the purpose is to find informative genes indicative of the cancer status for a general population. As far as we know, all of the LOOCV accuracy reported in the literature for these 9 data sets used all the samples to select informative genes. We followed the same routine so that our results can be compared to those reported in the literature.

First, we consider the average accuracy for each algorithm across all cancers. Using the informative genes selected with our method, the performance of SVM has significantly improved from average accuracy 91.19% to 97.69% across nine datasets. LDA and QDA cannot be applied directly to high dimensional dataset since they require estimation of the high dimensional covariance matrices whose estimate is not accurate with limited sample sizes. With the genes selected by our method, BMSF-LDA and BMSF-QDA performed very well with average accuracy 94.82% and 94.67% across nine datasets. The top three methods with highest average accuracy among all those in Additional file 1: Table S2 are BMSF-SVM, BMSF-LDA and BMSF-QDA. The k-TSP method used to give the best performance among those reported in literature for these nine datasets. It has 2.66%, 2.81%, and 5.68% more classification errors on average than BMSF-LDA, BMSF-QDA, and BMSF-SVM, respectively.

For individual cancer dataset, the BMSF either outperforms or has comparable performance to the best result reported in the literature. For the CNS data, it can be seen from Figure 2 that BMSF-LDA and BMSF-QDA have equivalent accuracy as k-TSP, which are slightly higher than BMSF-NB and BMSF-SVM. The rest of the classifiers have much lower accuracy. For the Colon data, BMSF-SVM has much better performance than the other algorithms. For the DLBCL data, BMSF-SVM have comparable top performance to k-TSP and TSP, followed by BMSF-LDA,SVM, BMSF-QDA, and sign-PAM that have slightly lower accuracy. For the GCM data, all the classifiers with the BMSF selected variables have better accuracy than those using PAM and TSP. In this case, SVM also gives good result. For the Leukemia dataset, BMSF -LDA, BMSF-SVM and SVM have the best accuracy followed by BMSF-QDA. For the Lung data, BMSF-SVM gives the best accuracy and BMSF-NB, SVM, sign-PAM, mul-PAM and k-TSP fall slightly below. For the Prostate1 data, BMSF-SVM is ahead of the other algorithms; BMSF-LDA and TSP have comparable performance that is slightly higher than BMSF-QDA; BMSF-NB is not as good as the rest of the algorithms. For the Prostate2 data, the four algorithms using BMSF selected variables obviously outperform the rest of the algorithms with a big margin. BMSF-SVM, BMSF-LDA, BMSF-QDA and BMSF-NB had accuracies 98.86%, 94.32%, 90.91% and 89.77%, respectively, while direct application of SVM only gave an accuracy of 76.14% and the highest accuracy reported in the literature is 81.25%. For the Prostate3 data, BMSF-NB/QDA/SVM and SVM all achieved 100% accuracy; BMSF-LDA, mul-PAM, k-TSP, and TSP have equivalent performance.

Beyond accuracy comparison, we also report the number of genes used in each classifier in Table 4. Excluding the TSP method that can only consider pairs, the number of genes selected by our method is among the top two smallest sets over all data.

**Table 4:** Number of genes used in the classifiers for gene-expression datasets

**Method**	**CNS**	**Colon**	**DLBCL**	**GCM**	**Leukemia**	**Lung**	**Prostate1**	**Prostate2**	**Prostate3**
TSP*	2	2	2	2	2	2	2	2	2
k-TSP*	10	2	2	10	18	10	2	18	2
SVM/NB ^‡^	7129	2000	7129	16063	7129	12533	12600	12625	12626
PAM*	4	15	17	47	2296	9	47	13	701
Sumdiff/ mul/sign –PAM^†^	286	80	286	642	286	502	504	506	506
HBE^Ѕ^	-	-	6	-	4	-	10	-	-
BBF-SVM^Þ^	-	20	5	-	-	-	13	-	-
Random forest (GeneSrF)	11	3	3	186	4	12	2	4	3
BMSF-SVM/NB/ LDA/QDA^ϒ^	3	7	6	32	3	8	8	11	2

*Results obtained in Tan et al. [3]^†^Results obtained in Chopra et al. [12].

^Þ^Results obtained in Zhang and Deng [10]^Ѕ^Results obtained in Dagliyan et al. [4]^‡^ Results obtained using all genes.

^ϒ^Refers to each of the classifiers using our selected genes.

### Average of absolute correlation among genes

**Figure 3:**
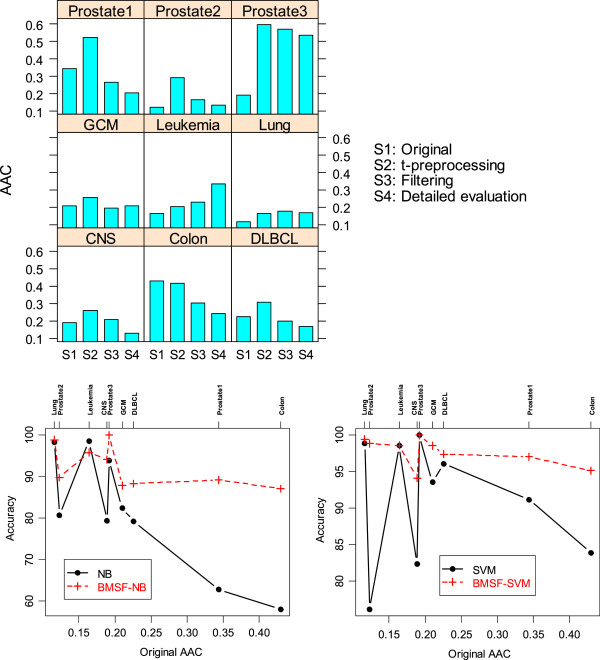
**Average of absolute correlation (AAC) at each stage and its relationship with NB, SVM.** The top panel gives the AAC for each dataset. ‘Original’ refers to the entire dataset; ‘filtering’ refers to the stage after the procedures in Section 5.1; ‘Detailed evaluation’ refers to the stage at the end of Section 5.2. The bottom panels show the relationship between the AAC on the original dataset with NB, BMSF-NB, SVM, and BMSF-SVM classifiers. The original AAC appears to be reversely related to the accuracy of NB. The relationship of the original AAC with SVM is not obvious. BMSF-NB and BMSF-SVM are much less influenced by the original AAC.

In general the average of absolute correlation (AAC) among all genes in each dataset is not very high due to the fact that the majority of genes contain a lot of random noises. The genes retained after prescreening with the t-test typically have higher AAC than that for the entire set of genes. This is because many co-regulated genes displaying changes during the cancer progression were retained after the prescreening. Our algorithm tends to select gene sets with low redundancy. This can be seen in the top panel of Figure 3. The AAC after the filtering steps (see Section 5.1) and fine evaluation steps (see Section 5.2) is much smaller than the AAC after the t-test for all but the Leukemia, Lung, and Prostate3 datasets. The AAC for Leukemia increased at the end of our algorithm compared to that after the t-test. For the Lung and Prostate3 datasets, the AAC did not change much. Incidentally, these three datasets are the only ones that the best LOOCV accuracy is 100% and there are other algorithms that could have comparable performance as our algorithm does.

The bottom 2 panels of Figure 3 shows the relationship between the AAC in the original datasets and NB, SVM classifiers. In can be seen that the LOOCV accuracy of NB tends to be lower for datasets with higher original AAC. BMSF-NB is not as influenced by the original AAC on accuracy as the NB. For the SVM classifier, original AAC is related to its performance and there may be other factors affecting its performance. Regardless of the magnitude of the original AAC, BMSF-SVM produced consistently much better results.

### Variability of results and joint effects of genes selected from different runs of BMSF

**Figure 4:**
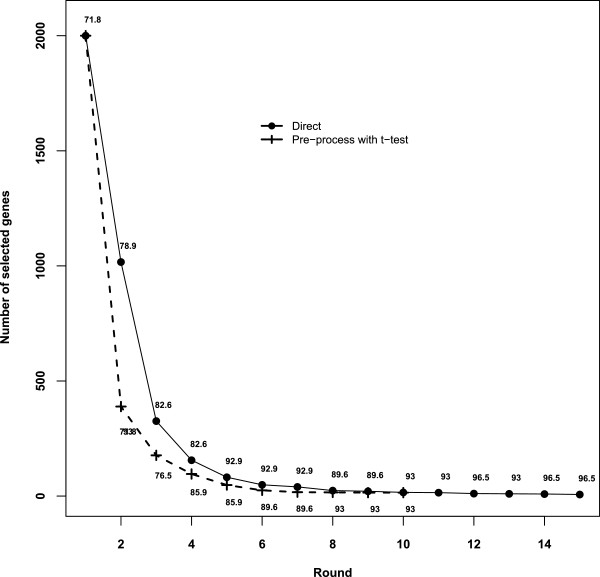
**The change in the number of selected genes in each round.** The values labeled are the best MCC.

Even though we did gene prescreening with t-test for consistency with most methods in the literature, our result indicated that the prescreening was not necessary. Figure 4 depicts the changing pattern in the number of genes and the corresponding best MCC values at each round of filtering steps in Sections 5.1 - 5.2. The second and third columns correspond to the direct application of our method on the colon cancer data, while the last two columns correspond to the case with prescreening via t-test. Even though the prescreening with the t-test can save some time by drastically reducing the number of genes at the beginning, it did not result in better MCC values. Further fine evaluation with the procedure in Section 5.2 led to a final list of 7 genes with LOOCV accuracy 95.16% for the case with prescreening (Table 5) and 6 genes with LOOCV accuracy 98.39% for the direct application (Table 1). Genes T48041, M19311, X57206 and T57882 deemed unimportant by the univariate t-test were among the final list of informative genes that gave excellent LOOCV classification accuracy. This suggests that the synergistic effects of these genes gave more differentiating power than their individual effect.

**Table 5:** Selected genes from Colon dataset after screening by t-test

**Accession number**	**p-value of t-test**	**p-value of paired t-test**
Z50753	4.7E-06	5.2E-12
H67764	0.01822	5.4E-15
H17434	0.00514	1.7E-14
R88740	0.02422	0.00022
R36977	0.00119	2.6E-18
R81170	0.02326	2.6E-18
U14631	0.00248	0.00002

The paired t-test was conducted according to the method described in section "Importance ordering and significance of the selected genes".

The list of informative genes may be different in separate runs of our algorithm. This is because the random matrix **X** generated in Section 5.1 may contain different combinations of genes to be included for filtering. This, however, should not significantly affect the accuracy of the algorithm as there are several rounds of random matrix generation and filtering to include as many combinations as possible. To support this argument, we obtained 3 lists of informative genes for the Leukemia dataset by running our algorithm multiple times. The average accuracies are reported in Table 6. The three lists of informative genes are totally different but the LOOCV accuracies are very close suggesting that this dataset has high redundancy. Biological interpretation of such phenomenon would be that the selected genes are not the only genes that have gone through changes during cancer progression. Two lists of the genes could each be a partial list of all genes showing differences between the cancer statuses. On the other hand, our algorithm is meant to select a parsimonious set of genes to achieve high LOOCV classification accuracy. The selection process is not designed to exhaustively find all genes that exhibit difference for the two categories.

**Table 6:** Results from three runs of the leukemia dataset

**Run**	**Selected genes**	**LOOCV accuracy**
1	X95735_at, Y07604_at, D26156_s_at	98.61
2	U82759_at, X95735_at, Y07604_at	97.22
3	M23197_at, U77604_at, M28170_at	100

**Figure 5:**
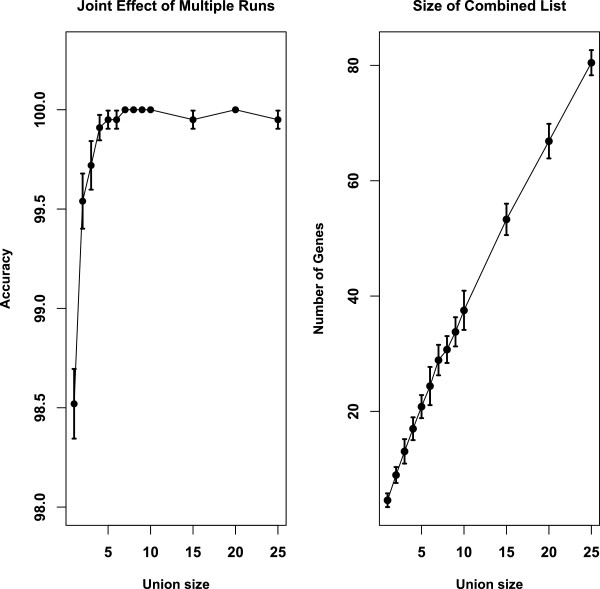
**Joint effect of informative genes from multiple runs of the leukemia dataset.** The left panel gives the LOOCV accuracy +/− standard error from 30 runs using the combined list of genes. The right panel gives the number of genes in the combined list +/− standard deviation from 30 runs. In both plots, the number of lists being combined is in the horizontal axis.

From a biological point of view, it might be interesting to know whether the combination of the gene lists from different runs can produce similar classification accuracy. Studying this can also infer the stability of BMSF algorithms in terms of commonly selected genes in different runs, and the joint effects of the genes on classification accuracy. As an example, we considered multiple runs of BMSF-SVM on the leukemia data. We performed 30 runs (see Additional file 1: Table S3) and randomly choose the results from *k* runs, where *k* = 1, 2,…, 10, 15, 20, 25 is the union size. The informative genes from the *k* runs are combined to make a new list of informative genes and LOOCV classification with this combined list of genes is conducted. This procedure of random selection of *k* runs and classification was repeated 30 times to assess the results of the joint effect. As the union size increases, the size of the combined list of genes increases. The average LOOCV accuracy from the 30 random selections of single run is 98.52% with standard deviation 0.96%. Thirty random selections of two runs yield an average accuracy 99.54% with standard deviation 0.76%. For 30 random selections of three runs, the average accuracy using the combined genes from three runs is 99.72% with standard deviation 0.67%. For combined genes from more than four runs, the average accuracy from 30 random selections is at least 99.91% with standard deviation less than 0.35%. The results are summarized in Figure 5. The left plot in Figure 5 gives the average LOOCV accuracy from 30 classifications using the 30 combined lists from random selections of *k* runs. The standard errors are indicated with the error bars. The right plot in Figure 5 gives the number of genes in the combined list as a function of the union size *k*. The error bars in the right plot gives the standard deviation from the 30 random selections. In summary, as the union size *k* increases, the average accuracy increases and the standard deviation of the accuracy decreases.

### Comparison with 11 other variable selection methods from RankGene and mRMR

To compare with other variable selection methods, we choose variables with BMSF and 11 other variable selection criteria available through RankGene at http://genomics10.bu.edu/yangsu/rankgene/ and mRMR at http://penglab.janelia.org/proj/mRMR/. RankGene provides eight ranking criteria including t-statistic, twoing rule, information gain, gini index, max minority, sum minority, sum of variances, and one-dimensional support vector machine. These criteria rank genes based on their capability to distinguish between the classes. The user is required to specify the number of genes to be selected. mRMR conducts minimum redundancy maximum relevance feature selection [8,17]. MID and MIQ are two versions of mRMR highly recommended by Peng et al. [17] and Ding and Peng [8]. The mRMR can be used alone with a pre-specified number of variables to select or used with a classification algorithm to choose the set of variables that minimize cross-validation error.

MID and MIQ discretize the expression data into intervals for both noise reduction and ease of estimation of the mutual information. It has been reported in Ding and Peng [8] that discretization leads to better classification accuracy in mRMR than directly classifying the continuous expression data.

Different discretization schemes were reported to give consistent results ([8]; http://penglab.janelia.org/proj/mRMR/FAQ_mrmr.htm#Q5.3) when the expression values are transformed into 2 or 3 states by comparing to *μ*±*kσ* for k ranges from 0.5 to 2, where μ and σ are gene specific mean and standard deviation respectively. We take *k=1* in our experiments such that the expression values greater than *μ+σ* were discretized into state 1; values between *μ-σ* and *μ+σ* are transformed to state 0; and values less than *μ-σ* are transformed to state −1.

As RankGene does not provide a list of the informative genes as BMSF and mRMR do, we consider to base our comparisons on the LOOCV classification accuracy of four algorithms (LDA, QDA, SVM, NB) using a pre-specified number of genes selected from each criterion. The error rate of mRMR tends to decrease as the number of genes increases [8,17]. Though BMSF and mRMR with a classification algorithm both report a list of informative genes, the numbers of genes are different. mRMR tends to need more genes to have low error rates. We set the number of genes for RankGene and mRMR to be the number selected according to BMSF on each of the nine cancer datasets. This allows us to examine if the space of informative genes generated by BMSF can be more easily classified than those generated by mRMR or RankGene.

**Figure 6:**
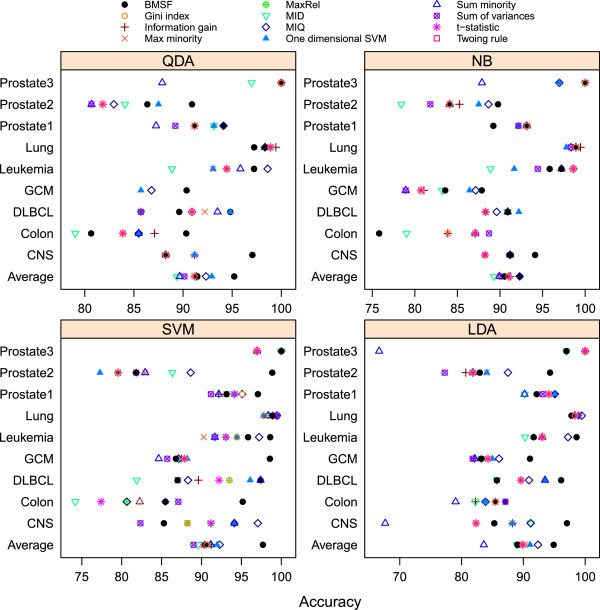
**Comparison of different variable selection methods for the same classification algorithm.** For each of the classification algorithms (LDA, QDA, SVM, NB), identical number of genes are selected for each cancer dataset by BMSF and 11 other variable selection criteria (the number of genes used is according to BMSF). The LOOCV accuracy is presented in the dotplot, in which the coordinate of a point in the horizontal axis indicates the accuracy. A point located to the right represents higher accuracy than a point located to the left. In most of the cases, the algorithms with variables selected by BMSF reach the highest LOOCV accuracy. For the GCM data, the variables selected by the eight criteria from RankGene and MaxRel cannot perform QDA due to rank deficiency. So the average accuracy for QDA is calculated over the other datasets for fair comparison.

The LOOCV accuracy for each of the four classification algorithms (LDA, QDA, SVM, and NB) is presented in the dotplot in Figure 6 (the numerical accuracy is in the Additional file 1: Table S4). In the plot, the coordinate of a point in the horizontal axis indicates the accuracy. A point located to the right represents higher accuracy than a point located to the left. In most of the cases, the algorithms with variables selected by BMSF reach the highest LOOCV accuracy. An explanation that BMSF outperforms mRMR is that mRMR criteria maximize the average of all mutual information values between individual variable and class and minimize the average mutual information between two feature variables. If one variable is selected at a time in incremental search, it is shown in Peng et al. [17] that mRMR is equivalent to maximizing the dependency between the features and the target class (Max-Dependency). In reality, however, multiple variables may be selected at the same time. In such case, joint effects among multiple variables are not taken into account in mRMR. As a result, the list of selected variables for any pre-specified number is not the most efficient feature set to distinguish the classes among methods that allow multiple variables to be selected at a time. This is the case in our comparison.

It has been a common belief that a wrapper type feature selector can yield high classification accuracy for a particular classifier and less generalization of the selected variables on other classifiers. BMSF does not have such limitation as the superiority of BMSF is not restricted to the SVM that was wrapped with the algorithm for variable selection. For all four classification algorithms we considered (LDA, QDA, SVM, and NB), BMSF consistently outperforms the other variable selection criteria on each cancer data. The variables selected by BMSF yield comparable relative performance for different classifiers. This suggests that BMSF has the combined high accuracy property of wrapper feature selectors and generality property of filter type variable selectors.

### Comparison with random forest and SVM-RFE

In this subsection, we report comparison with the random forest and SVM-RFE algorithms. We apply the SVM-RFE with linear kernel. The algorithm starts with all the features and eliminates one feature with the least squared weight at each step until all the features are ranked. In a consistent way to the treatment by [21] for application of SVM-RFE, the original datasets are normalized by subtracting the mean of the corresponding gene vector from each gene’s expression data and then dividing by the corresponding standard deviation.

SVM-RFE does not perform feature selection. Instead, it only provides a list of ranked genes and the user need to decide the number of genes to be selected. For this reason, we cannot do a general fair comparison with it because our method decides the number of genes to be selected. So we only report a brief comparison, in which the SVM-RFE was applied to the entire data and a list of ranked genes are obtained. We then plotted the LOOCV accuracy of LDA, NB, and SVM classifiers using *k* top ranked genes from SVM-RFE for *k* = 2, …, 150. The accuracy of SVM in general increases as more genes are included in the model. The accuracies of LDA and NB may fluctuate drastically or decrease as the number of genes increases. This suggests that the gene ranking by SVM-RFE is SVM specific and may not generalize well to NB or LDA. The plotted accuracy curve is an oracle situation which assumes the number of genes is a known priori. Without knowing the number of genes to be used, additional variability will add to the LOOCV accuracy. For convenience of comparison, the LOOCV accuracy of the LDA, NB, and SVM classifiers using the genes selected by BMSF are shown in the plot with diamond-shaped points. The horizontal coordinates of the points are set to be the number of genes selected by BMSF. At each point location, the value on the curve of the same color as the diamond point is the SVM-RFE accuracy if SVM-RFE uses the same number of genes as BMSF. These values are given in Additional file 1: Table S4. With the same number of genes as BMSF, the QDA classifier yielded comparable average accuracy for SVM-RFE and BMSF; the SVM and NB classifiers had less average accuracy with the SVM-RFE genes than with the BMSF genes; the LDA classifier had slightly better accuracy with the SVM-RFE genes than with the BMSF genes. We emphasize that the users are not recommended to interpret the results discussed here strictly since in practice it is necessary to estimate the number of genes in order to use the SVM-RFE which will lead to additional variability on the results.

We also compare with the random forest gene selection algorithm GeneSrF provided by Diaz-Uriarte [49]. GeneSrF was first applied to each dataset to select genes followed by an application of LDA, QDA, NB, and SVM classifiers using the selected genes with LOOCV. Since the GeneSrF algorithm also automatically determines the number of genes to be selected, we can compare BSMF and GeneSrF on a fair basis. The results are also reported in Additional file 1: Table S4. The LDA classifier with the GeneSrF selected genes has lower accuracy than the same classifier used with the BMSF selected genes for 8 out of the 9 datasets. The exception is the Lung cancer data, for which the BMSF and GeneSrF genes have comparable performance. The average accuracy of the LDA classifier over the 9 datasets with BMSF genes is 94.82, while that for the GeneSrF genes is 88.23. Similarly, the QDA and SVM classifiers show better accuracy with the BMSF selected genes than with the GeneSrF selected genes for most of the datasets. The NB classifier showed comparable performance for the BMSF and GeneSrF genes. These results are expected since it is reported in Díaz-Uriarte and Alvarez de Andre’s 2006 that the random forests feature selection has comparable performance to KNN and SVM. From our earlier comparison in Section 2.2 and Additional file 1: Table S2, we know that BMSF outperforms SVM in most of the datasets. In terms of the number of genes selected from an entire dataset, GeneSrF reported less numbers for 5 of the datasets than BMSF and both GeneSrF and BMSF selected no more than 11 genes in these 5 datasets. In the remaining 4 datasets, GeneSrF selected more genes than BMSF. In particular, GeneSrF selected 186 genes while BMSF only selected 32 genes for the GCM data.

Upon the request of a reviewer, we also conducted comparison with repeated 10-fold cross validation. SVM-RFE is excluded from the comparison since it does not determine the number of genes. We compare the gene selection using the BMSF and random forest by examining at the average accuracy from multiple runs. For each random partition of the data into 10-folds, 9 of the subsets are used as trainning and the rest is used as the test data. BMSF and random forest (GeneSrF) are used for gene selection and SVM, NB, LDA, QDA are used to build model with the training data and predict the class of the test data. The accuracy from each run of 10-fold CV is the proportion of correctly classified samples. We conducted 10 random partitions and reported the mean and standard deviation of the 10-fold CV accuracy from the 10 runs in Table 7. The results show similar pattern to the LOOCV accuracy discussed earlier. That is, the NB classifier using the BMSF has comparable performance to that using GeneSrF selected genes. The SVM and LDA classifiers with the BMSF selected genes show much better performance than the same classifiers using the GeneSrF selected genes. The QDA classifier with the genes selected by BMSF has only slightly better results than those selected by the GeneSrF.

**Table 7:** Average and standard deviation of 10-fold CV accuracy from 10 runs

	**BMSF- SVM**	**GeneSrF- SVM**	**BMSF- NB**	**GeneSrF- NB**	**BMSF- LDA**	**GeneSrF- LDA**	**BMSF- QDA**	**GeneSrF- QDA**
CNS	94.11(1.38)	88.23(0)	92.05(2.79)	91.17(0)	96.47(1.86)	83.23(3.93)	95.88(2.05)	NA(NA)
Colon	94.35(2.04)	78.22(2.18)	87.41(1.98)	86.93(1.93)	87.41(1.48)	81.45(0.85)	89.51(1.9)	81.12(1.08)
DLBCL	98.57(1.29)	88.44(2.48)	88.96(0.68)	88.57(0.54)	96.23(0.41)	85.71(1.36)	94.15(1.1)	90(0.62)
GCM	98.07(0.61)	92.07(0.9)	87.42(0.76)	84.17(0.24)	91.14(0.66)	77.53(1.41)	90.25(0.93)	NA(NA)
Leukemia	98.33(0.58)	96.66(0.97)	96.25(0.93)	94.58(0.43)	98.33(0.58)	93.19(0.43)	97.63(0.67)	94.58(0.78)
Lung	99.11(0.64)	98.95(0.4)	98.39(0.31)	98.34(0)	97.79(0.26)	98.34(0)	97.56(0.46)	98.34(0.26)
Pros1	96.76(0.8)	92.64(1.24)	89.6(0.82)	93.33(0.62)	95.49(0.5)	90.98(1.01)	93.23(0.85)	91.56(0.68)
Pros2	97.38(1.2)	83.75(2.27)	90(1.17)	85.11(1.46)	95.34(1.13)	85.45(0.71)	90.11(2.27)	83.63(1.09)
Pros3	98.48(1.59)	93.93(2.47)	99.69(0.95)	99.69(0.95)	96.66(0.95)	93.93(0)	100(0)	96.66(1.72)

BMSF and random forest (GeneSrF) are used for gene selection and SVM, NB, LDA, QDA are used to build model with the training data and predict the class of test data.

## Discussion

In this article, we proposed a new variable selection algorithm BMSF that can select a small set of feature variables taking into account variable interactions to provide highly accurate classification of the samples. The BMSF method automatically conducts multiple rounds of filtering and guided random search in the large feature subset space and reports the final list of informative genes. Though the variable selection process is wrapped with SVM, the variables selected have general applicability to multiple classification algorithms. SVM, LDA, QDA and NB achieved superior LOOCV classification accuracy with the selected variables from BMSF compared to 11 other variable selection criteria.

Different runs of BMSF may produce different lists of informative genes. This phenomenon corresponds to the fact that there are many possible prognostic genes. Our goal is to find a minimal set of such genes that the combination of them can well differentiate the cancer status of the patients. Additional genes that are important for sample classification can be obtained by carrying out multiple runs of BMSF. For example, the U82759_at selected from the second run of BMSF on the Leukemia data encode HoxA9 (an Oncogene) that has a higher level of expression in the samples from AML patients than in the ALL patients [4]; the M23197_at gene selected on the third run encodes CD33 antigen (differentiation antigen) that is a membrane protein. CD33 has been reported to be a target for antibody-based anti-leukaemic therapies as about 85%-90% of acute AML cases are considered to be CD33 positive [50]. Consequently, the resulting genes found from different runs can be combined to provide a larger gene set signature. Our experiments showed that the joint effects of the genes from different runs typically increase the classification accuracy.

It may be interesting to combine multiple variable selection methods for better results. We comment that BMSF can be used along with other methods as long as the operation does not violate the principle of each method. Consider Prediction Analysis of Microarrays (PAM) by Tibshirani et al. [5] for an example. PAM is not a classification algorithm alone. Instead, it performs both variable selection and classification. A thresholding parameter Δ needs to be estimated with cross-validation. Each parameter value Δ corresponds to a set of genes selected for classification. If we apply PAM to each cancer dataset with the variables already selected by BMSF, PAM would conduct variable selection again using all samples within the subset of genes selected. This violates the principle of PAM when only a small number of variables remain in the model because PAM employs the thresholding idea to identify sparse signals out of a large amount of noise (the test statistic value from each of a large number of irrelevant variables corresponds to noise and those from the few important variables are the signals; after variable selection by BMSF, the total number of variables may not be large enough for the thresholding idea to be legitimate). However, PAM may be applied first to the original dataset since it tends to select many more variables than BMSF. For example, the PAM application in Leukemia dataset with the best Δ value yields a model with more than 1000 variables. In such a case, BMSF can still be applied to further reduce the number of variables.

As with all feature selection results in microarray data, the variables selected may or may not be a subset of cancer progression signature. Future validation of clinical relevance of the selected genes through multiple external cancer cohorts composes another line of work (see for example [51-53]). They mainly address how to assess a gene set signature’s prognostic value of a predefined size relative to random gene sets. Boutros et al. [51] also presented a nonlinear Steepest Gradient Descent (greedy forward selection) algorithm to identify prognostic gene signatures from their 158-gene RT-PCR training dataset of 147 patients. As is discussed in the introduction the greedy search strategy with wrapper method is not tractable in current setting with high dimensional feature space. Our work in this article provides a useful tool in this regard.

Currently, this software was only implemented to perform two-class classification. In recent years, SVM has been extended to perform multi-class classification and regression. Further directions along this line can consider extending the method to perform variables selection when there are multiple classes or when the response variable is continuous. We anticipate such extension to be very helpful to improve the accuracy of multi-class cancer classification and genome wide association study.

## Conclusions

In summary, considering the discriminating power of a gene after adjusting for the joint effect of many other genes and their possible interactions can improve the classification accuracy. We provided an effective algorithm BMSF to carry out the search of important variables in the high dimensional feature space allowing interactions of a large number of genes. The algorithm automatically reports a list of very small number of selected genes that renders high classification accuracy for multiple classifiers. Our method not only overcomes the difficulty associated with the search schemes of traditional wrapper methods in large dimensional search space but also has good generality for the genes selected. This is confirmed by testing our method on the 9 benchmark binary class gene expression datasets all related to human cancers.

## Methods

Consider gene expression data with G genes from N tissue samples. Denote the G genes as {g_1_, g_2_, …, g_G_}, and the N tissue samples as {t_1_, t_2_, …, t_N_}. The objective is to assign a class label for each tissue sample t_i_ based on the observed expression data matrix D from all genes. D is a NxG matrix with the i^th^ column represents the expression values for gene g_i_ from all tissue samples, and j^th^ row represents the expression values for all genes from tissue j. G is typically much larger than N, especially with high density microarrays. In this study, we concentrate on the two-class case, in which one class may be cancerous tumor and the other class is normal, or the two classes may refer to cancer subclasses such as different stages of a cancer. Denote the two classes as positive (+) and negative (−). The variable selection process seeks to identify the genes such that the diagnosis of a tissue can be performed via modeling the expression data of these genes, i.e., corresponding columns of the matrix D. It has reached consensus that not all genes are necessary to build a good diagnosis classifier; and classifiers with less number of genes but with equivalent or better accuracy is preferred for ease of interpretation and further study of the marker genes.

### Fast filtering

#### First round of filtering

Since the modeled effect of one gene’s interaction with other genes may change as the members of a gene set vary, we perform multiple rounds of filtering to exclude the large number of irrelevant genes. Before filtering, we perform a 5-fold cross-validation (CV) to predict the class labels using all genes and record the initial Matthew’s correlation coefficient (MCC) defined as follows [54]:

$$Ø=\frac{TP\times TN-FP\times FN}{\sqrt{\left(TP+FN\right)\times \left(TN+FP\right)\times \left(TP+FP\right)\times \left(TN+FN\right)}}$$

where TP is the number of true positives, TN the number of true negatives, FP the number of false positives and FN the number of false negatives. If any of the four sums in the denominator is zero, the MCC value *Ø* is set to zero since the numerator is zero. The values of MCC is in the range of [-1, 1] with value 1 indicating a perfect prediction and 0 an average random prediction. MCC is used instead of the accuracy defined as the proportion of correctly classified subjects because MCC is more stable even if the classes are of very different sizes. Denote this initial MCC value as μ_0_.

Steps 1 – 3 below constitute the first round of filtering.

Step 1 Specify genes to be included to perform guided random search. As is known, tumor development typically undergoes through a complicated process that involve many genes. To describe the combination of gene sets to be considered for modeling, we generate a matrix X with dimensions KxG with entries being either 1 or 0, representing whether the gene in that column is included in the modeling or not. We take K to be a large even number such as 500, 400, etc. The columns of X were generated by permuting the entries of a K-dimensional vector that contains K/2 ones and K/2 zeros. That is, there are equal numbers of ones and zeros in each column of X such that the same gene is included in the modeling K/2 times and excluded from the modeling K/2 times. Different columns of X are different random permutations of the K-dimensional vector. Each row of X is a G-dimensional vector with value 1 representing inclusion of the genes designated by corresponding columns, and 0 representing exclusion of the genes in corresponding columns. Each row of X defines a gene set to be considered for model training in the next step. Different rows of X give different gene sets formed through including a combination of a random number of genes. For convenience, we call X the inclusion scheme matrix.

Step 2 Evaluation of gene set contributions. For i=1, …, K, denote the number of ones in the ith row of X as pi and the locations of these ones in the vector as li1, li2, …, lipi. The values of pi may be different for different rows. The expression data from genes gli1, gli2, …, glipi and the class information of all subjects will be used in cross-validation. We consider 5-fold CV of the subjects using SVM, in which the subjects are partitioned into five folds via stratified sampling and the expression data and class labels from four folds of the subjects are employed to train the SVM model. The obtained model is then applied to predict the class label for the remaining one-fold of the subjects. After prediction is carried out for each fold, we calculate the Matthews correlation coefficient using predictions for all subjects. Repeat this operation for i=1, …, K. The obtained MCC values for all rows give us an initial idea of the contribution from each of the randomly formed gene set. Denote the vector of MCC values calculated for all rows of X as *Ø*_0_.

Step 3 Assess individual gene’s contribution after adjusting for contributions from other genes. A gene may be related to the cancer status on its own or through its interaction with other genes. To assess the relative contribution of gene gi taking into account of its possible interaction with other genes, we compare the two cross-validated prediction performances, one with the gene included and one with the gene excluded from a gene set. We consider the gene sets defined in the inclusion scheme matrix X. For the ith gene, this is performed as follows.

1) Obtain matrix **X**_**i**_ by changing all the ones in i^th^ column of **X** to zero and all the zeros in that column to one while keeping the remaining columns of **X** unchanged.

2) Train a SVM regression model m_0_ using *Ø*_0_ as the response vector and **X** as the design matrix for independent variables.

3) Predict the value of the response variable for each row of **X**_**i**_. Denote the K-dimensional vector of all predicted response values as *Ø*_1_.

4) Form paired data Z_0_ and Z_i,_ each of K dimension. The k^th^ entry z_0k_ of Z_0_ and z_ik_ of Z_i_ are defined as;

$$\begin{array}{cc} \;z_{0k} & =\left\{\begin{array}{c} k^{\mathrm{th}}value\;of\;{Ø}_{o\;}if\;\chi_{\text{ki}}=0\\ k^{\mathrm{th}}value\;of\;{Ø}_{i}\;if\;\chi_{\text{ki}}=1, \end{array}\right)\\ \;z_{\mathrm{ik}} & =\left\{\begin{array}{c} k^{\mathrm{th}}value\;of\;{Ø}_{o\;}if\;\chi_{\text{ki}}=1\\ k^{\mathrm{th}}value\;of\;{Ø}_{i}\;if\;\chi_{\text{ki}}=0\text{,} \end{array}\right) \end{array}$$

where x_ki_ is the entry at the k^th^ row and i^th^ column of **X**. In other words, Z_0_ collects all those *Ø*_0_ and *Ø*_1_ entries such that the i^th^ gene is excluded in the modeling. Z_i_, on the other hand, collects all those *Ø*_0_ and *Ø*_1_ entries such that the i^th^ gene is included in the modeling. The entries of Z_0_ and Z_i_ are arranged in the same order as the rows of **X.** Such arrangement assures that the Z_0_ and Z_i_ entries are paired in the sense that except for the difference of inclusion or exclusion of the i^th^ gene, the conditions of all other genes are held identical. Comparing Z_0_ and Z_i_ would give us an idea of how significant the i^th^ gene contributes to explain the variations in the class prediction performance conditional on various combinations of other genes included in the model.

5) Calculate the average value of entries in Z_0_ and Z_i_ and denote them as ${\overset{Â¯}{z}}_{0}$ and ${\overset{Â¯}{z}}_{i}$, respectively. If ${\overset{Â¯}{z}}_{0}$ >${\overset{Â¯}{z}}_{i}$, then excluding the i^th^ gene tend to give better class prediction performance measured by MCC. For crude filtering at this stage, we permanently remove the i^th^ gene from further modeling if ${\overset{Â¯}{z}}_{0}$ >${\overset{Â¯}{z}}_{i}$ holds.

Perform 1) – 5) for all genes by letting i=1, 2, …, G. This finishes one round of filtering.

We comment that only one cross-validated training is performed for each row of the **X** matrix in the entire first round filtering. The total number of training is K. For each **X**_**i**_, the trained SVM model is used for prediction K times. After all **X**_**i**_ , i=1, 2, …, G, are considered, the total number of predictions is in the order of KG. This fully makes use of the advantage of the SVM that is much faster in prediction than training.

Another comment we make is about the number of columns in the inclusion scheme matrix **X.** In Step 1, even though each row of the inclusion scheme matrix **X** includes G/2 features on average, we did not use these G/2 features alone to eliminate features. A gene included in a row that does not contribute to the cancer status variation with genes in that row may contribute through interaction with genes not included in this row. That is, a gene in one gene set may not show marginal or interaction effect but it may have strong interaction effect with genes in another gene set. Therefore, we do not eliminate features based on one random subspace. Instead, different rows of the inclusion scheme matrix **X** include different gene sets (each of size G/2 on average). The feature elimination is done by considering the effect of a feature in different feature sets in Step 3. When all the rows of matrix **X** are used in the evaluation of the effect of a single gene in Step 3, many gene combinations are considered such that all G genes are included (instead of G/2 genes). In classical ensemble methods, the elimination of a feature is only based on the model with the features included in the subspace that the ensemble classifier is built. In such case, a feature could be eliminated by mistake if the important feature combination including this feature is not in the subspace. Our complicated way of filtering hopefully could reduce the chance of mistakenly eliminating an important feature. The price to pay for taking into consideration of all G features in evaluation of a single feature is the computational challenge with a large number of features specified by each row of **X**. This, however, is not a concern with the LIBSVM because LIBSVM is fast when there are a large number of features and small sample size (LIBSVM is slow when the number of features is small and the sample size is large).

### Further rounds of filtering

Our experience tells that roughly about half of the genes are excluded after the first round filtering. Let G_1_ be the number of genes that are kept after the first round filtering. Now we consider using only the expression data for these G_1_ genes and the class labels in cross validation with SVM to calculate current MCC value μ_1_. If μ_1 <_ μ_0,_ the filtering can stop as the performance with the reduced gene set is worse than the performance before the genes reduction. If μ_1 >_ μ_0,_ it is necessary to further reduce the number of irrelevant genes and we perform a second round filtering by repeating the procedures in 5.1 with data from these G_1_ genes.

In general, after each round of filtering, we calculate the cross validated MCC performance for SVM using all the genes kept at the end of previous round filtering. If the new MCC value is greater than the previous MCC value, the filtering continues to the next round. Otherwise, the filtering stops.

The number of random combinations K (i.e., the row dimension of **X**) should not be too small such that enough cases of interaction of a gene with other genes can be considered. The K is taken to be 500 for the first round filtering, 450 for the second round, 400 for the third round, etc. and the lower bound for K is 300. For the colon cancer dataset (Alon et al., 1998), we performed fifteen rounds of filtering with K=500, 450, 400, 350 for the first four rounds, and K=300 for the remaining eleven rounds. The total number of SVM models trained is equal to 502 +451 +401 +351 +301 x11 = 5016 , among which 5001 models are support vector classification with sample size being the same as the number of original sample, and 15 models are support vector regression with sample sizes being in the range of 500 to 300. Only 7 genes are kept after the 15 rounds of filtering. These 7 genes can be studied in detail for fine evaluation. Setting K gradually decreasing corresponds to the reduced number of independent variables in the model as the variable elimination continues because less numbers of combinations are needed. This will make the program run faster and faster as it proceeds.

Iterative filtering is also used in the random forest feature selection algorithm of Díaz-Uriarte and Alvarez de Andre’s (2006). Fitting one subspace decision tree may take less time than one round of our filtering, but the number of trees in each decision forest required to have stable values of variable importance is in the magnitude of 2000 to 5000 (Díaz-Uriarte and Alvarez de Andre’s 2006; [25]). In addition, the percent of features to be eliminated from each decision forest is a parameter that requires user’s input. The default percentage in Díaz-Uriarte and Alvarez de Andre’s (2006) is set to be 20%, which eliminates 20% of the features from each fitted decision forest. Since each decision forest is restricted in a subspace, the total number of iteratively fitted decision forests is very large in order to eliminate majority of features. On the other hand, our algorithm starts with all G features and eliminates about half on each round of filtering. So the total number of rounds is about log_2_(G), which is much smaller than the number of decision forests that need to be fitted.

### Fine evaluation of candidate genes retained from earlier filtering

After the filtering procedure in 5.1 – 5.2, the number of genes has been dramatically reduced. In the 9 datasets we considered, the number of retained genes is 20 or less for 8 of the datasets. The remaining dataset had 36 genes retained. For these small numbers, fine evaluation of each gene taking into account its interaction with other genes can be performed more accurately. We perform the following backward elimination procedure to achieve this goal. Initialize Ω to be the collection of all genes retained after the filtering in 5.1 – 5.2. Ω will be updated as the algorithm below proceeds.

(a) Denote k to be the number of candidate genes in Ω.

(b) With all k candidate genes in Ω, obtain the MCC value with 10-fold CV using support vector machine classification. Denote the MCC value as *Ø*^(k)^.

(c) Leave out the i^th^ gene and use the remaining k-1 genes in 10-fold CV with SVM classification to obtain the Matthews correlation coefficient *Ø*_-i_. Perform this for all i=1, 2, …, k.

(d) If max{*Ø*_i_, 1≤ i ≤ k} <*Ø*^(k)^, skip e) and f) and go to step g).

(e) Let j be the gene in the candidate list in Ω such that *Ø*_-j_ = max{*Ø*_-i_, 1≤ i ≤ k}. Remove the j^th^ gene from the candidate list Ω and change the variable k to have value k-1.

(f) Repeat a) – e).

(g) Report all the genes that are in Ω. These are the final list of informative marker genes to be used for cancer classification.

The stopping criterion in (d) sometimes may produce some unnecessary computation when max{*Ø*_-i_, 1≤ i ≤ k} and *Ø*^(k)^ are very close. An alternative is to treat the number of features in the final classifier as a tuning parameter or simply define a threshold of the importance for the purpose of feature selection. For example, the entire fine evaluation step can be replaced with SVM-RFE, in which the number of features in the final classifier will be left as a tuning parameter. This alternative direction may be able to speed up the fine evaluation process. But adding additional tuning parameter also raises a question of how to estimate this parameter. It is an open question and additional study will be required to evaluate how well it works.

### Importance ordering and significance of the selected genes

Relative importance of each informative gene in the final list can be further ordered through multi-round elimination. Denote k_0_ as the number of selected informative genes in the final list.

(i) Let m = k_0_ and define a buffer set ß that contains all genes to be ordered. The initial members of ß contain all genes in the final list. Initialize М to be a null vector to store the genes in the order of importance.

(ii) For t=1, …, m, remove the t^th^ gene in ß and use the remaining m-1 genes in 10-fold CV with SVM classification to obtain the Matthews correlation coefficient *Ø*_-t_. Perform this for all t=1, 2, …, m.

(iii) Let q be the gene in ß such that *Ø*_-q_ = max*Ø*_t_, 1≤ t ≤ m}. Remove the q^th^ gene from ß and make it the first element of М by shifting the other elements in М accordingly. Change variable m to have value m-1.

(iv) Repeat steps (i) – (iii) until only one gene is left in ß. Move this gene to М so that this gene is the first element in М and other genes are shifted accordingly.

At the end of step (iv), the genes in М are ordered. The most important gene is the first element in М and the importance of other genes reduces as the position of a gene moves upward.

Alternatively, it might be desirable to give the significance of the selected genes. This can be achieved with the procedure in I) – IV) below.

(i) If the number of informative genes k_0_ is no more than 9, we can consider all combinations of the informative genes and form a matrix **X**_0_ with k_0_ columns and 2^k0^ rows. The elements of **X**_0_ are zeros or ones indicating whether the genes designated by the column numbers are excluded or not for modeling. The total number of rows is no more than 2^9^ =512.

(ii) If the number of informative genes k_0_ is more than 9, we generate a matrix **X** with K rows and k_0_ columns using these k_0_ genes as described in Step 1 of Section 5.1.1. Further, append **X** with additional k_0_+1 rows, the first of which is composed of all ones and the last k_0_ rows form an identity matrix. Denote the resulting (K+ k_0_+1) by k_0_ matrix as **X**_0_.

(iii) Perform Steps 2 and 3 of Section 5.1.1 using matrix **X**_0_ with the original expression data for all the k_0_ informative genes and class labels for all subjects. By the end of this operation, paired data Z_0_ and Z_i_ are obtained for each informative gene, one representing prediction performances without this gene, and the other one representing the performances with the gene while all other informative genes are considered in the modeling.

(iv) For each informative gene, carry out a paired t-test using the paired data obtained in III) and report the *p*-value.

Each row of the identity matrix appended to **X** in II) serves as a univariate evaluation of the contribution of each gene. Such evaluation ignores the possible co-regulation of genes and their interactions. Consequently, they can only be used along with combinations of other genes provided by other rows of **X** to give a fair evaluation of each gene’s contribution.

The entire procedure described in 5.1 – 5.3 uses the Matthew’s Correlation Coefficient of SVM as the objective function to guide the search for optimal gene set. We have avoided the computational infeasibility associated with exhaustive search by repeatedly considering random combinations of genes. As the support vector machine is the tool to study the relationship between the class labels and the informative genes, the possible nonlinear dependence on each gene and its interaction with other genes are captured. The evaluation process described in section 5.3 may employ a different classifier since the number of informative genes tends to be small at that stage. However, the process in earlier sections relies on a classifier that is able to handle a large number of independent variables. K-nearest neighbor (KNN) algorithm also can perform classification with a large number of independent variables. However, since this algorithm barely differentiates among different genes in the sense that each gene in the nearest neighborhood of a point contributes equally in making class assignment, we prefer not to use KNN to guide the search.

### Software and Data

The BMSF Matlab code used in this article and the data sets with the selected genes are available in the zipped file Additional file 2. The NCBI links for the 9 cancer datasets used in this article are also given in this zipped file.

## Competing interests

The authors declare that they have no competing interests.

## Authors’ contributions

HZ, ZY and ZD developed the algorithm, HZ and HW performed the analyses for nine datasets, HW and HZ drafted the manuscript, ZD and HZ designed the software, ZY and MC provided discussion and revised the manuscript for this study. All authors have approved the final version of the manuscript.

## Supplementary Material

Additional file 1 **This file contains Supplementary Tables S1 - S4.** Supplementary Table S1 reports the Accession number, name, and putative function for selected genes in each data set. Supplementary Table S2 gives the comparison of LOOCV accuracy on nine cancer data sets for BMSF with results reported in literature. Supplementary Table S3 list the selected genes from 30 separate runs of BMSF on Leukemia data. Supplementary Table S4 reports the LOOCV accuracy of BMSF, random forest (GeneSrF), SVM-RFE, and 11 other variable selection criteria from RankGene and mRMR. The same number of genes (determined by BMSF) is used for all criteria except for random forest, which automatically determines the number of genes to be used. Click here for file

Additional file 2 **The BMSF Matlab code and datasets with selected genes are included in this file.** The NCBI links for the cancer datasets are also included. Click here for file
