# Supplementary material for: Improving accuracy for cancer classification with a new algorithm for genes selection
Source: BMC Bioinformatics. 2012 Nov 13;13:298. doi: 10.1186/1471-2105-13-298 (PMC3562261; doi:10.1186/1471-2105-13-298)
Supplement: Additional file 2 — The BMSF Matlab code and datasets with selected genes are included in this file. The NCBI links for the cancer datasets are also included. [file 1471-2105-13-298-S2.pdf]

Additional file 2: The BMSF Matlab code and datasets

Improving Accuracy for Cancer Classification with a New Algorithm for Genes Selection by Zhang et al.

The matlab code for BMSF along with an example and instructions are in the compressed file '**bmsf.zip**'. Matlab2009 should be installed prior to application of the BMSF algorithm.

Related Datasets

The nine cancer datasets are publicly available. The PubMed links are provided below.

- CNS (Pomeroy et al, 2002) http://www.ncbi.nlm.nih.gov/entrez/query.fcgi?cmd=retrieve&db=pubmed&list_uids=11807556
- Colon (Alon et al, 1998) http://www.ncbi.nlm.nih.gov/entrez/query.fcgi?cmd=Retrieve&db=pubmed&dopt=Abstract&list_uids=10359783
- DLBCL (Shipp et al, 2002) http://www.ncbi.nlm.nih.gov/entrez/query.fcgi?cmd=retrieve&db=pubmed&list_uids=11786909
- GCM (Ramaswamy et al, 2001) http://www.ncbi.nlm.nih.gov/entrez/query.fcgi?cmd=Retrieve&db=pubmed&dopt=Abstract&list_uids=11742071
- Leukemia (Golub et al, 1999) http://www.ncbi.nlm.nih.gov/entrez/query.fcgi?cmd=Retrieve&db=pubmed&dopt=Abstract&list_uids=10521349
- Lung (Gordon et al, 2002) http://www.ncbi.nlm.nih.gov/entrez/query.fcgi?cmd=Retrieve&db=pubmed&dopt=Abstract&list_uids=12208747
- Prostate1 (Singh et al, 2002) http://www.ncbi.nlm.nih.gov/entrez/query.fcgi?cmd=retrieve&db=pubmed&list_uids=12086878
- Prostate2 (Stuart et al, 2004) http://www.ncbi.nlm.nih.gov/entrez/query.fcgi?cmd=Retrieve&db=pubmed&dopt=Abstract&list_uids=14722351
- Prostate3 (Welsh et al, 2001) http://www.ncbi.nlm.nih.gov/entrez/query.fcgi?cmd=Retrieve&db=pubmed&dopt=Abstract&list_uids=11507037

Data sets with the selected genes are in the file '**selected gene-dataset.zip**'. Each row corresponds to one sample. The cancer classes are in the last column.
